# Genetically raised serum bilirubin levels and lung cancer: a cohort study and Mendelian randomisation using UK Biobank

**DOI:** 10.1136/thoraxjnl-2020-214756

**Published:** 2020-08-27

**Authors:** Laura Jane Horsfall, Stephen Burgess, Ian Hall, Irwin Nazareth

**Affiliations:** 1 Department of Primary Care & Population Health, UCL, London, UK; 2 MRC Biostatistics Unit, University of Cambridge Institute of Public Health, Cambridge, Cambridgeshire, UK; 3 BHF Cardiovascular Epidemiology Unit, Department of Public Health and Primary Care, Cambridge University, Cambridge, Cambridgeshire, UK; 4 Division of Respiratory Medicine, University of Nottingham, Nottingham, Nottinghamshire, UK

**Keywords:** lung cancer, oxidative stress, smoking cessation, tobacco and the lung

## Abstract

**Background:**

Moderately raised serum bilirubin levels are associated with lower rates of lung cancer, particularly among smokers. It is not known whether these relationships reflect antioxidant properties or residual confounding.

**Objective:**

This study aimed to investigate potential causal relationships between serum total bilirubin and lung cancer incidence using one-sample Mendelian randomisation (MR) and UK Biobank.

**Methods:**

We instrumented serum total bilirubin level using two variants (rs887829 and rs4149056) that together explain ~40% of population-level variability and are linked to mild hereditary hyperbilirubinaemia. Lung cancer events occurring after recruitment were identified from national cancer registries. Observational and genetically instrumented incidence rate ratios (IRRs) and rate differences per 10 000 person-years (PYs) by smoking status were estimated.

**Results:**

We included 377 294 participants (median bilirubin 8.1 μmol/L (IQR 6.4–10.4)) and 2002 lung cancer events in the MR analysis. Each 5 μmol/L increase in observed bilirubin levels was associated with 1.2/10 000 PY decrease (95% CI 0.7 to 1.8) in lung cancer incidence. The corresponding MR estimate was a decrease of 0.8/10 000 PY (95% CI 0.1 to 1.4). The strongest associations were in current smokers where a 5 μmol/L increase in observed bilirubin levels was associated with a decrease in lung cancer incidence of 10.2/10 000 PY (95% CI 5.5 to 15.0) and an MR estimate of 6.4/10 000 PY (95% CI 1.4 to 11.5). For heavy smokers (≥20/day), the MR estimate was an incidence decrease of 23.1/10 000 PY (95% CI 7.3 to 38.9). There was no association in never smokers and no mediation by respiratory function.

**Conclusion:**

Genetically raised serum bilirubin, common across human populations, may protect people exposed to high levels of smoke oxidants against lung cancers.

**Key messages**

**What is the key question?**

- Is there evidence that moderately raised serum bilirubin, a purported antioxidant, can protect against lung cancer and does the relationship differ according to smoking status?

**What is the bottom line?**

- Current and former cigarette smokers genetically predisposed to raised levels of serum total bilirubin have lower rates of lung cancer with the association strongest in current heavy smokers. There was no relationship between genetically instrumented or observed bilirubin levels for never smokers.

**Why read on?**

- This is the first study to support a potential causal association between serum bilirubin and the incidence of lung cancer in cigarette smokers.

## Introduction

Lung cancer is the leading cause of cancer death globally due to the high incidence and poor prognosis.^1^ Risk assessment for lung cancer in primary care settings relies on self-reported smoking status, which can be challenging to quantify accurately. Identifying simple blood biomarkers for lung cancer, comparable with cholesterol in cardiovascular disease, could improve the cost-effectiveness of new screening programmes^2^ and potentially lead to new treatments for oxidative stress-mediated diseases.

Following the natural death of red blood cells, a series of reactions initiated by the haem oxygenase enzymes one and two (HMOX1/HMOX2) generates the yellow pigment bilirubin. At first, bilirubin is water insoluble and is transported in the blood serum to the liver where it is converted to a soluble (conjugated) form for elimination by the enzyme uridine diphosphate-glucuronosyltransferase 1–1 (UGT1A1). Bilirubin levels are frequently tested in primary healthcare settings to assess liver function and guide prescribing decisions for drugs including statins. Various lines of evidence, such as the high frequency of *UGT1A1* alleles causing mild hereditary hyperbilirubinaemia (Gilbert’s syndrome; OMIM#143500), has led to speculation that moderately raised levels may have a physiological benefit for humans.^3–5^ Investigations using blood serum, tissue cultures and animal models suggest that bilirubin has potent antioxidant properties that may help protect respiratory tissues against oxidative stress.^5–8^ For example, the enzymes that generate bilirubin (biliverdin reductase, HMOX1) are found at their highest concentrations in respiratory tissues 9 and a recent network analysis of the effect of cigarette smoke on the lung tissue of mice identified dramatic increases in bilirubin production implying a protective role.^10^ Population-based cohort studies have also linked moderately raised serum bilirubin levels with lower rates of respiratory diseases and lung cancer with the strongest associations in cigarette smokers.^11–14^ Although these observations are consistent with an endogenous antioxidant function for bilirubin, the relationships with lung cancer could reflect residual confounding or reverse causation. For instance, liver diseases, infections, acute events such as heart attacks, certain drugs and environmental exposures can influence bilirubin levels.^11 15 16^

Mendelian randomisation (MR) is an approach that, with important assumptions, could address certain limitations of observational studies of bilirubin and provide more robust support of a causal mechanism.^17^ First, the random allocation of alleles during gamete formation and conception should balance observed and unobserved confounders across genetic groups. Second, genotype status is unaffected by disease processes and reverse causation is unlikely. Finally, genotyping is less prone to random error and regression dilution bias. Here, we present a large-scale MR examining the relationship between genetically raised serum bilirubin and lung cancer in adults participating in UK Biobank. We also report the cross-sectional associations between bilirubin and respiratory function as a potential mediator of the relationship with lung cancer.

## Methods

### Data source

The UK Biobank Resource is a prospective cohort study of over 500 000 participants aged 40–69 years and recruited between 2006 and 2010 from different regions of the UK.^18^ Further information on UK Biobank is provided in the online supplementary methods and at the website (https://www.ukbiobank.ac.uk/).

10.1136/thoraxjnl-2020-214756.supp1 Supplementary data

### Study design

Initially, to verify previous studies, we analysed the observational relationship between serum total bilirubin levels measured at baseline and incident lung cancer. We also examined the cross-sectional relationship between observed serum bilirubin and FEV_1_. Second, using MR approach, we estimated the potential causal relationships between bilirubin levels and these outcomes using individual-level data. The study protocol was approved by UK Biobank in July 2018 (ID: 5167) and adequacy of sample size was checked using online tools (http://cnsgenomics.com/shiny/mRnd/).

### Inclusion/exclusion criteria

After excluding people no longer wishing to participate, the total number of participants available for analysis was 502 527. We applied several genetic exclusions including those recommended by UK Biobank, outliers for genotype missingness or excess heterozygosity, sex aneuploidy and sex discordance (n=2200). We used a published algorithm to retain unrelated participants^19^ (n=39 642) and finally restricted the sample to ‘white British’ participants using a combination of self-reported ethnic identity and the results of an existing principal components analysis available in the dataset (n=88 341).^20^ Participants entered the cohort on the date they attended the research centre and were censored at the earliest date of lung cancer diagnosis, loss to follow-up, death or end of the follow-up period. The most recent date of complete monitoring for incident cancers at the time of analysis was 31 March 2016 for England and Wales and 31 October 2015 for Scotland. Participants with a history of lung cancer at the time of recruitment were excluded from the primary analysis of lung cancer incidence (n=527).

### Exposures

Blood samples were collected at baseline from all participants, and serum total bilirubin was assayed by photometric colour (Beckman Coulter AU5800). Genome-wide association studies (GWAS) have repeatedly identified *UGT1A1* at 2q37 as the major locus underlying serum bilirubin levels across human populations.^21–23^ A GWAS of British subjects found the single nucleotide polymorphism (SNP) rs887829 explained 39%–43% of the variation in bilirubin levels.^24^ This SNP is in almost complete linkage disequilibrium (r2=0.99) with the functional repeat-length polymorphism of the *UGT1A1* promoter region underlying Gilbert’s syndrome.^3 25^ Bilirubin GWAS have consistently replicated another signal at 12p12.^22 25 26^ The nonsynonymous SNP rs4149056 (Val174Ala) of the Solute Carrier Organic Anion Transporter Family Member 1B1 (*SLCO1B1)* is a lead causal SNP in this region and transports bilirubin from blood into the liver. Both rs887829 and rs4149056 were included on the microarrays and we used these variants to instrument bilirubin levels. Missing genotypes were replaced with imputed data where available.^20^ Based on known substrates for these enzymes and searches of online GWAS repositories, we did not expect any important confounding of the genetic relationships by other mechanisms (pleiotropy).

### Outcomes

The primary outcome is incident lung cancer recorded following study recruitment. Prevalent and incident cancer diagnoses in UK Biobank are provided by The Health & Social Care Information Centre for participants residing in England and Wales, and the NHS Central Register for participants residing in Scotland. These national cancer registries obtain information from a range of sources including hospitals, treatment centres, hospices and nursing homes, private hospitals, general practices, death certificates and Hospital Episode Statistics. Underlying (primary) cause of death is also provided from central registers. Diagnoses and causes of death are coded using the International Classification of Disease (ICD) version 9 and 10. Malignant neoplasms of the trachea and bronchus (ICD10: C33-C34) are the cancers where smoking has the strongest pathophysiological role and highest attributable risk at >70%, and we selected these cancers as our primary outcome.^27^ A self-reported cancer diagnosis is also available and used in addition to ICD codes to identify participants’ cancer history.

Other risk factors for lung cancer including FEV_1_ and family history of lung cancer, and comorbidity for COPD or emphysema are potentially on the causal pathway between bilirubin antioxidant activity and lung cancer. We therefore examined these separately as secondary outcomes in a series of cross-sectional analyses (online supplementary methods). Oxidative stress is thought to have a pathophysiological role in many age-related diseases, and as further supplemental analyses, we also explored relationships with mortality from any cause and cancer mortality.

### Covariates

Basic characteristics and known predictors of lung cancer were included in analyses to improve the precision of the estimates.^17 28^ Depending on the outcome, these included age, calendar year, genetic sex, recruitment centre, height, weight and self-reported smoking status. We also included the top 40 principal genetic components to account for any remaining population substructure. Other variables we examined in supplemental analyses of a subsample with complete data on all covariates included passive smoking, occupational exposure to smoke, antioxidant supplements (vitamin C, vitamin E and β-carotene), social deprivation, air pollution (NO_2_ and PM2.5), waist circumference and liver blood tests (alkaline phosphatase, alanine aminotransferase and gamma-glutamyl transferase).

### Interactions

Studies have reported negative associations with bilirubin and associated *UGT1A1* genotypes that are strongest in current and heavy smokers, which is consistent with endogenous antioxidant properties.^12 29 30^ Therefore, we examined interactions with self-reported smoking behaviour and categorised participants into never, former and current smokers. To examine smoking intensity more closely, we further divided smokers who indicated they currently or previously smoked tobacco on most or all days as ever light-moderate smokers (1–19 cigarettes per day) or heavy smokers (20 or more cigarettes per day). Pack-years of smoking had previously been calculated for participants who smoked tobacco on most or all days and also reported their smoking duration.

### Statistical analyses

#### Observational relationships

Serum bilirubin levels were divided into sex-specific quintile categories to describe the relationships with other covariates. We used multivariable Poisson regression with age as the time scale to estimate the lung cancer incidence rate ratios (IRRs) with robust SEs per 5 μmol/L increase in serum total bilirubin (entered as a continuous variable). We also explored non-linear relationships with serum bilirubin and age by applying cubic spline-interpolation and selecting the transformation that minimised the Akaike and Bayesian information criteria (AIC/BIC). The goodness of fit to the Poisson distribution was checked using the deviance statistic and by fitting negative binomial models and comparing outputs. We ran an overall analysis and then added multiplicative interaction terms to derive the smoking-specific IRRs. Multiplicative interaction terms on the ratio scale are difficult to interpret for non-linear models such as Poisson. Also, the risk of lung cancer varies dramatically by smoking status and estimates on the incidence rate (additive) scale are also useful for interpretation. We therefore calculated the margins of response as adjusted incidence rates and rate differences at different levels of bilirubin (1–30 μmol/L) while holding all other variables at their observed values. SEs for marginal effects were calculated using the delta method. Further details on methods and various sensitivity and cross-sectional analyses of potential mediators are reported in the online supplementary methods.

#### Genetically instrumented relationships

We calculated the crude lung cancer incidence rates across genotypes stratified by smoking status. For the *UGT1A1* variant (rs887829), we estimated the relationship between homozygosity for the T allele linked to Gilbert’s syndrome and lung cancer on the rate ratio scale and incidence rate difference scale. We then combined the effects of rs887829 and rs4149056 on bilirubin levels to estimate the IRRs for lung cancer per 5 μmol/L increase genetically predicted bilirubin using one-sample MR and the two‐stage predictor substitution (2SPS) method.^17^ Further details are reported in the online supplementary methods.

All statistical analyses were conducted using Stata V.16.1 (Stata Corporation, College Station, Texas, USA) except for the exclusion of relatives, which was done using R (V.3.5.1).

## Results

After excluding outlier values (n=414), serum total bilirubin levels were available for 357 802 participants with a median value of 8.1 μmol/L (IQR 6.4–10.4) (table 1). There were 2.5 million person-years of follow-up, 1917 incident cases of lung cancer diagnosed after recruitment, 15 532 deaths from any cause and 768 participants were lost to follow-up for various reasons including leaving the UK. Men and women with low serum bilirubin were heavier, more likely to smoke and live in socially deprived areas (table 1). A diagnosis of lung cancer or COPD/emphysema prior to recruitment was twice as common the lowest bilirubin quintile versus the highest (table 1).

**Table 1:** Baseline characteristics of UK Biobank participants by sex-specific quintiles of serum bilirubin levels

Covariates	Total	Sex-specific quintile categories of serum total bilirubin (μmol/L)					P value*	Missing data (%)
Women		1.1	5.6	6.7	7.9	9.8–41		
Men		1.1	6.7	8.4	10.0	12.5–41		
	N=357 802	N=71 230	N=71 514	N=71 793	N=71 580	N=71 685		
Sex	165 817 (46.3%)	33 089 (46.5%)	33 057 (46.2%)	33 205 (46.3%)	33 241 (46.4%)	33 225 (46.3%)		
Age at recruitment (IQR)	58.9 (51.4–64.0)	58.3 (51.1–63.7)	59.2 (51.8–64.0)	59.3 (51.9–64.1)	59.2 (51.5–64.1)	58.5 (50.5–63.9)	<0.0001	
Weight (kg)	78.3 (15.9)	79.7 (16.4)	78.8 (15.9)	78.3 (15.7)	77.7 (15.6)	77.3 (15.5)	<0.0001	986 (0.3)
Height (cm)	168.8 (9.2)	167.9 (9.2)	168.5 (9.2)	168.9 (9.2)	169.2 (9.2)	169.6 (9.2)	<0.0001	714 (0.2)
BMI	27.4 (4.7)	28.2 (5.1)	27.7 (4.8)	27.4 (4.7)	27.1 (4.6)	26.8 (4.5)	<0.0001	1107 (0.3)
Waist circumference (cm)	90.4 (13.5)	92.4 (13.7)	91.0 (13.4)	90.3 (13.3)	89.5 (13.3)	88.8 (13.3)	<0.0001	576 (0.2)
Smoking status							<0.0001	
Never	194 558 (54.4%)	34 687 (48.7%)	38 066 (53.2%)	39 326 (54.8%)	40 390 (56.4%)	42 089 (58.7%)		
Former	125 965 (35.2%)	24 529 (34.4%)	25 115 (35.1%)	25 746 (35.9%)	25 554 (35.7%)	25 021 (34.9%)		
Current	36 059 (10.1%)	11 739 (16.5%)	8089 (11.3%)	6490 (9.0%)	5382 (7.5%)	4359 (6.1%)		
Prefer not to say	1220 (0.3%)	275 (0.4%)	244 (0.3%)	231 (0.3%)	254 (0.4%)	216 (0.3%)		
Pack years of smoking (IQR)†	19.5 (10.0–32.5)	22.8 (12.2–36.0)	20.2 (10.5–33.5)	18.8 (9.8–31.9)	18.0 (9.0–30.5)	16.5 (8.6–28.5)	<0.0001	
Occupational smoke exposure	94 720 (26.5%)	22 933 (32.2%)	19 566 (27.4%)	18 262 (25.4%)	17 343 (24.2%)	16 616 (23.2%)	<0.0001	
Exposure to smoke at home	32 620 (9.1%)	6707 (9.4%)	6546 (9.2%)	6494 (9.0%)	6404 (8.9%)	6469 (9.0%)	0.002	
Antioxidant supplements	97 340 (27.2%)	19 770 (27.8%)	19 595 (27.4%)	19 510 (27.2%)	19 477 (27.2%)	18 988 (26.5%)	<0.0001	
Nitrogen dioxide air pollution (µg/m^3^); 2010 (IQR)	25.6 (21.0–30.4)	25.9 (21.3–30.6)	25.5 (21.0–30.4)	25.4 (20.9–30.3)	25.4 (20.9–30.3)	25.4 (20.8–30.3)	<0.0001	4817 (1.4)
Particulate matter air pollution (µg/m^3^) (PM2.5); 2010 (IQR)	9.88 (9.23,10.49)	9.93 (9.28,10.54)	9.89 (9.23,10.50)	9.86 (9.21,10.48)	9.86 (9.21,10.48)	9.85 (9.20,10.46)	<0.0001	30 282 (8.5)
Townsend deprivation index (IQR)	−2.4 (−3.7–0.1)	−2.1 (−v3.6–0.6)	−2.3 (−3.7–0.1)	−2.4 (−3.8 to -0.1)	−2.4 (−3.8 to -0.2)	−2.5 (−3.8 to -0.2)	<0.0001	431 (0.1)
History of lung cancer	527 (0.15%)	152 (0.21%)	115 (0.16%)	85 (0.12%)	99 (0.14%)	76 (0.11%)	<0.0001	
Family history of lung cancer	46 093 (12.9%)	9510 (13.4%)	9374 (13.1%)	9157 (12.8%)	9167 (12.8%)	8885 (12.4%)		
History of COPD/emphysema	8129 (2.3%)	2106 (3.0%)	1736 (2.4%)	1487 (2.1%)	1506 (2.1%)	1294 (1.8%)	<0.0001	
FEV_1_ (L)	2.9 (0.8)	2.8 (0.8)	2.8 (0.8)	2.9 (0.8)	2.9 (0.8)	2.9 (0.8)	<0.0001	86 032 (24)
FEV_1_ (L) ATS/ERS reproducibility	2.8 (0.7)	2.7 (0.7)	2.8 (0.7)	2.8 (0.7)	2.8 (0.7)	2.9 (0.7)	<0.0001	144 935 (41)
Lung cancer events	1917	548	418	356	328	267		
Person years (10 000)	252	50	50	50	51	50		
Incidence rate per 10 000 (95% CI)	7.6 (7.3–8.0)	10.9 (10.1–11.9)	8.3 (7.5–9.1)	7.1 (6.4–7.9)	6.5 (5.8–7.2)	5.3 (4.7–6.0)	<0.0001‡	

All continuous variables are mean values with ±1 standard or medians for skewed data if IQRs are specified.

*P values for sex-adjusted associations with log-transformed serum total bilirubin levels as the dependent variable.

†Previously calculated for 109 312 participants reporting to regularly smoke at least 1 cigarette/day and who also reported smoking duration.

‡Poisson model with lung cancer incidence as the dependent variable adjusted for sex.

ATS/ERS, Reproducibility of spirometry measurement using American Thoracic Society (ATS) and European Respiratory Society (ERS); BMI, body mass index; PYs, person-years.

**Figure 1:**
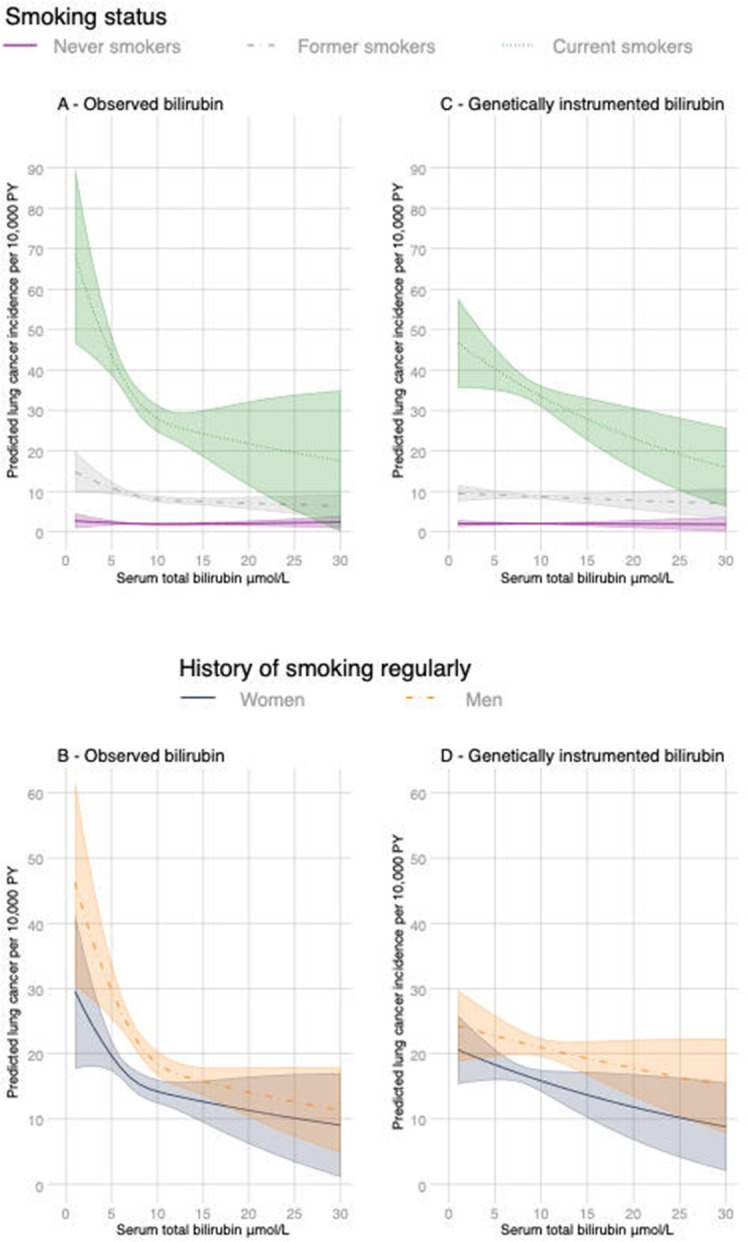
Margins of response by smoking status as adjusted lung cancer incidence rates with 95% CIs (shaded) at different levels of observed bilirubin (panels A and B) and genetically predicted bilirubin (panels C and D) while holding all other variables at their observed values. Variables included age, gender, calendar year, ethnicity (first 40 principal components) and recruitment centre. Predictions for observed bilirubin also include height and weight. Non-linear relationships were captured using cubic spline transformation with three knots placed at the 10th, 50th and 90th percentiles of bilirubin levels. Values with 95%CIs are reported in online supplementary table S3.

There was a disagreement between the Akaike and Bayesian information criteria on the functional form for bilirubin levels and we have therefore reported the estimates assuming a linear relationship with lung cancer (BIC) and a three-knot cubic spline transformation (AIC) (figure 1A and table 2). Assuming a linear relationship, each 5 μmol/L increase in adjusted serum bilirubin was associated with a 1.2 per 10 000 PY decrease in lung cancer (95% CI 0.7 to 1.8) (table 2 and online supplementary table S1). The strongest relationships were in current heavy smokers with an estimated decrease in lung cancer of 18.4 per 10 000 PY (95% CI 3.4 to 33) per 5 μmol/L increase in serum bilirubin (table 2, figure 1A and online supplementary table S1). The predicted incidence using the cubic-spline transformation and adjusting for confounders shows that the negative association for smokers is steepest at lower bilirubin levels (figure 1A). The independent associations between serum bilirubin and lung cancer remained after restricting the analysis to participants with a history of smoking at least one cigarette per day and adjusting for pack-years of smoking (table 2 and online supplementary table S1). Based on the predictive margins, the incidence of lung cancer in current smokers with a bilirubin level >17 μmol/L (often used to diagnose Gilbert’s syndrome) is around 35%–50% lower relative to a similar group of smokers in the lowest bilirubin quintile (figure 1 and online supplementary table S3). The AIC and BIC favoured a three-knot cubic spline transformation of bilirubin levels for participants with a history of smoking regularly (figure 1B). Including further covariates for a subset with complete data (1666 events) slightly attenuated the relationships (online supplementary figure S1). Serum bilirubin levels contributed to all models with spline interpolation (Wald test; p<0.0001).

**Table 2:** Observational relationships between mean serum total bilirubin and lung cancer incidence by smoking status in white British unrelated participants in UK Biobank

	Lung cancer events	PYs (10 000)	Lung cancer incidence rate per 10 000 PYs (95% CI)	Adjusted IRR per 5 μmol/L increase (95% CI)*	P value	Predicted incidence change in 10 000 PYs per 5 μmol/L increase (95% CI) *
Overall	1901	251	7.6 (7.2 to 7.9)	0.85 (0.80 to 0.92)	<0.0001	−1.2 (−1.8 to −0.7)
Never smokers	261	137	1.9 (1.7 to 2.1)	1.00 (0.87 to 1.15)	0.97	0.01 (−0.3 to 0.3)
Former smokers	888	88	10.1 (9.5 to 10.8)	0.87 (0.79 to 0.96)	0.0037	−1.2 (−2.1 to −0.4)
Current smokers	743	25	29.7 (27.6 to 31.9)	0.74 (0.63 to 0.86)	<0.0001	−10.2 (−15.0 to −5.5)
Prefer not to report	9	1	10.8 (5.6 to 20.7)			
Regular smokers (cigarettes per day) †						
* *Overall	1422	75	18.9 (17.9 to 19.9)	0.77 (0.70 to 0.84)	<0.0001	−5.0 (−6.8 to −3.2)
* *Former 1–19	187	24	7.7 (6.7 to 8.9)	0.95 (0.80 to 1.12)	0.52	−0.4 (−1.7 to 0.8)
Former ≥20	554	32	17.5 (16.1 to 19.0)	0.79 (0.69 to 0.90)	0.0006	−3.7 (−5.9 to −1.6)
Current 1–19	285	11	27.0 (24.0 to 30.3)	0.88 (0.70 to 1.13)	0.32	−4.2 (−12.3 to 3.9)
Current ≥20	341	7	51.7 (46.5 to 57.5)	0.76 (0.60 to 0.95)	0.021	−18.4 (−33.3 to −3.4)

*Adjusted for age, gender, calendar year, ethnicity (first 40 principal components), height, weight, recruitment centre and smoking status. Adjusted and unadjusted incidence rate ratios are reported in online supplementary table 1.

†Adjusted for pack-years, age, gender, calendar year, ethnicity (first 40 principal components), height, weight, recruitment centre. Participants currently smoking less than one cigarette per day at recruitment are excluded from the smoking subcategories but included in the overall analysis of regular smokers if they had formerly smoked one or more per day and it was possible to calculate pack-years.

IRR, incidence rate ratio; PYs, person-years.

**Figure 2:**
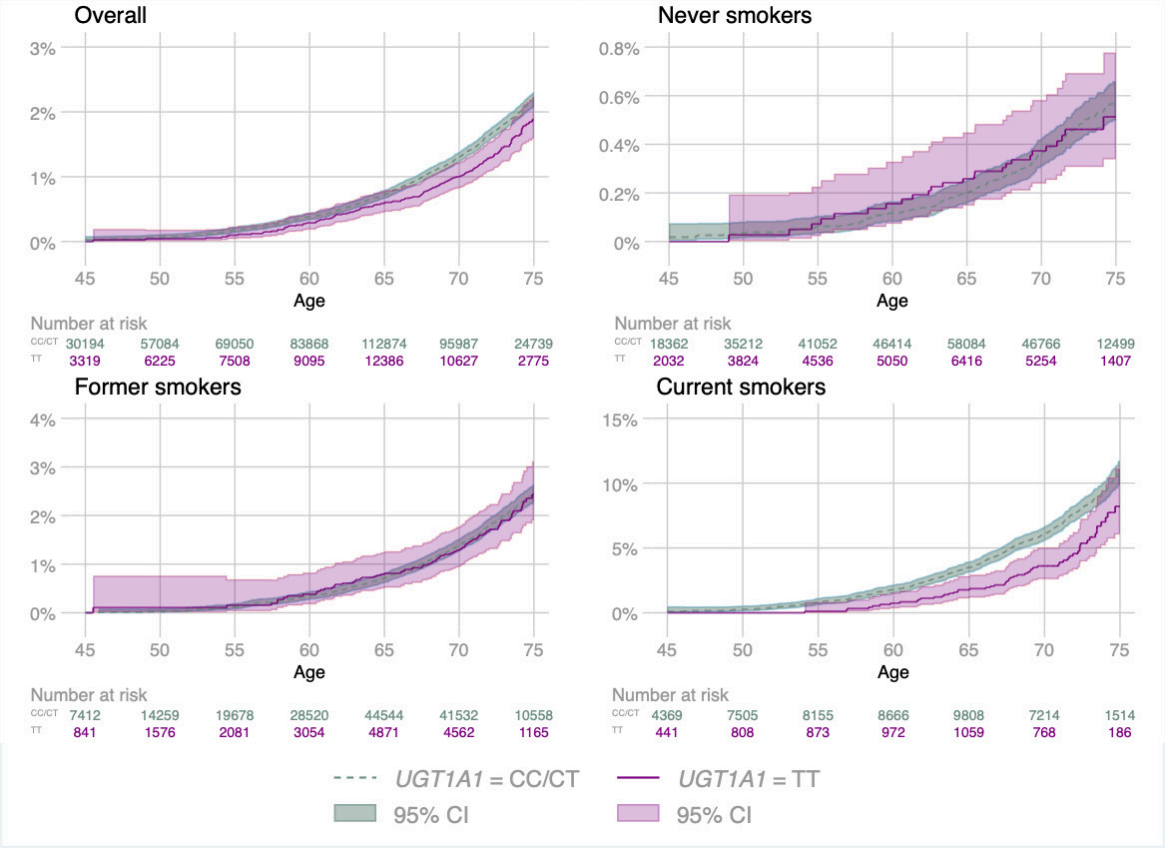
Nelson-Aalen estimate of the cumulative hazard function for the *UGT1A1* rs887829 genotype and lung cancer by smoking status.

The genetic analysis included 377 294 participants and 2002 lung cancer events. We confirmed that the selected SNPs were strongly associated with bilirubin levels with the *UGT1A1* rs887829 variant explaining 37% (F statistic=57 913) of the variability and a non-additive effect of *UGT1A1* rs887829 on bilirubin levels (table 3). In contrast to observed bilirubin levels (table 1), there were no associations between *UGT1A1* rs887829 status and the selected covariates (online supplementary table S2). Across most smoking strata, genotypes associated with the highest average bilirubin levels were associated with the lowest rates of lung cancer, particularly for current smokers (table 3, figure 2). In current smokers, the incidence of lung cancer in participants with the rs887829 TT genotype linked to Gilbert’s syndrome were estimated to be 11.1 per 10 000 PYs lower (95% CI 4.1 to 18.1) compared with the other genotypes with the association even stronger in current heavy smokers at 38.7 per 10 000 PYs lower (20.6 to 56.9) (table 4).

**Table 3:** Genotype frequency and association with serum bilirubin levels and crude lung cancer incidence rates for SNPs used in genetic instrumentation

SNP	Number (%)	Median serum total bilirubin (μmol/L) (IQR)	Lung cancer events	Lung cancer incidence per 10 000 PYs (95% CI)			
				Overall	Never smokers	Former smokers	Current smokers
***UGT1A1* rs887829**		R^2^=37%					
CC	177 209 (47.0)	7.2 (5.9–8.8)	960	7.7 (7.2 to 8.2)	1.9 (1.6 to 2.3)	10.3 (9.4 to 11.3)	30.2 (27.3 to 33.4)
CT	162 815 (43.2)	8.5 (6.9–10.6)	875	7.7 (7.2 to 8.1)	1.9 (1.6 to 2.3)	9.8 (8.9 to 10.8)	31.3 (28.2 to 34.7)
TT	37 270 (9.9)	15.7 (12.0–20.3)	167	6.4 (5.5 to 7.4)	1.8 (1.2 to 2.7)	9.4 (7.6 to 11.6)	21.4 (16.5 to 27.8)
	p value* <0.0001						
***SLCO1B1* rs4149056**		R^2^=1%					
TT	272 515 (72.2)	7.9 (6.3–10.2)	1458	7.6 (7.2 to 8.2)	1.8 (1.6 to 2.1)	10.2 (9.4 to 11)	30.1 (27.8 to 32.7)
TC	96 153 (25.5)	8.5 (6.7–10.9)	497	7.4 (6.7 to 8.0)	2.1 (1.6 to 2.6)	9.3 (8.2 to 10.6)	29.4 (25.6 to 33.8)
CC	8626 (2.3)	9.1 (7.3–11.6)	47	7.8 (5.8 to 10.3)	1.5 (0.6 to 3.7)	12.1 (8.2 to 17.8)	24.4 (14.7 to 40.6)
	p value* <0.0001						

*Kruskal-Wallis test.

PYs, person-years; SNP, single nucleotide polymorphism.

**Table 4:** Lung cancer incidence by *UGT1A1* rs887829 genotype and smoking status reported on the ratio and rate difference scales

*UGT1A1* rs887829/smoking status*	Lung cancer incidence per 10 000 PYs (95% CI)	IRR (95% CI)*	P value* for within smoking comparison	Lung cancer predicted incidence difference per 10 000 person years (95% CI)†	P value† for within smoking comparison
TT/never	1.8 (1.2 to 2.7)	1.0 (ref)	Ref	Ref	
CC or CT/never	1.9 (1.7 to 2.2)	1.1 (0.7 to 1.6)	0.82	0.1 (−0.6 to 0.8)	0.81
TT/former	9.4 (7.5 to 11.6)	4.2 (2.7 to 6.5)	Ref	Ref	
CC or CT/former	10.1 (9.4 to 10.8)	4.5 (3.2 to 6.7)	0.54	0.60 (−1.2 to 2.4)	0.54
TT/current	21.4 (16.5 to 27.8)	12.8 (8.0 to 20.4)	Ref	Ref	
CC or CT/current	30.1 (28.6 to 33.1)	18.6 (12.6 to 27.6)	0.0076	11.1 (4.1 to 18.1)	0.0019
Regular smokers (cigarettes per day)†					
TT/former 1–19	8.2 (5.4 to 12.6)	1.0 (ref)	Ref		
CC or CT/ former 1–19	7.7 (6.7 to 8.9)	0.9 (0.6 to 1.5)	0.78	−0.5 (−4.0 to 3.1)	0.79
TT/former ≥20	14.9 (11.3 to 19.7)	1.7 (1.0 to 2.9)	Ref		
CC or CT/former ≥20	17.4 (16 to 18.9)	2.0 (1.3 to 3.1)	0.33	2.0 (−2.0 to 6.2)	0.31
TT/current 1–19	24.7 (17.1 to 35.8)	4.1 (2.3 to 7.1)	Ref		
CC or CT/ current 1–19	27.3 (24.3 to 30.8)	4.5 (2.9 to 7.0)	0.60	3.6 (−9.0 to 16.1)	0.58
TT/current ≥20	26.8 (16.9 to 42.5)	4.5 (2.4 to 8.4)	Ref		
CC or CT/current ≥20	54.9 (49.5 to 61)	9.4 (6 to 14.6)	0.0023	38.7 (20.6 to 56.9)	<0.0001

*Rate ratios on the multiplicative (relative) scale and adjusted for age, gender, calendar year, ethnicity (first 40 principal components) and recruitment centre.

†Marginal effect on the additive (incidence) scale and adjusted for age, gender, calendar year, ethnicity (first 40 principal components) and recruitment centre.

IRR, incidence rate ratio; PYs, person-years.

The associations between genetically predicted bilirubin and lung cancer using the two SNPs was similar, although weaker than the observational relationships with levels measured at baseline (figure 1, table 5, online supplementary tables S1 and S3). The strongest associations were in current heavy smokers with each 5 μmol/L increase in genetically predicted serum bilirubin associated with a 23.1 per 10 000 PY decrease in incidence (95% CI 7.3 to 38.9). Adjusting for covariates made no material difference to the MR estimates (online supplementary table S1).

**Table 5:** Associations between genetically predicted serum total bilirubin and lung cancer incidence by smoking status in white British unrelated participants of UK Biobank

	IRR per 5 μmol/L increase (95% CI) *	P value	Estimated incidence change in 10,000PYs per 5 μmol/L increase (95% CI) *
Overall	0.90 (0.83 to 0.99)	0.023	−0.76 (−1.42 to −0.10)
Never smokers	0.98 (0.78 to 1.22)	0.87	−0.04 (−0.49 to 0.41)
Former smokers	0.95 (0.84 to 1.08)	0.40	−0.47 (−1.57 to 0.63)
Current smokers	0.83 (0.72 to 0.96)	0.011	−6.44 (−11.45 to −1.44)
Regular smokers (cigarettes per day)			
Overall†	0.89 (0.80 to 0.99)	0.028	−2.08 (−4.00 to −0.16)
Former 1–19	1.04 (0.81 to 1.33)	0.74	0.31 (−1.57 to 2.20)
Former ≥20	0.92 (0.79 to 1.08)	0.29	−1.38 (−3.94 to 1.18)
Current 1–19	0.94 (0.76 to 1.17)	0.57	−2.19 (−9.79 to 5.42)
Current ≥20	0.72 (0.58 to 0.90)	0.0037	−23.13 (−38.93 to −7.32)

*Adjusted for age, gender, calendar year, ethnicity (first 40 principal components), recruitment centre and smoking status. Adjusted and unadjusted incidence rate ratios are reported in online supplementary table S1.

†Adjusted for pack-years, age, gender, calendar year, ethnicity (first 40 principal components), recruitment centre in overall analysis of regular smokers.

IRR, incidence rate ratio.

**Figure 3:**
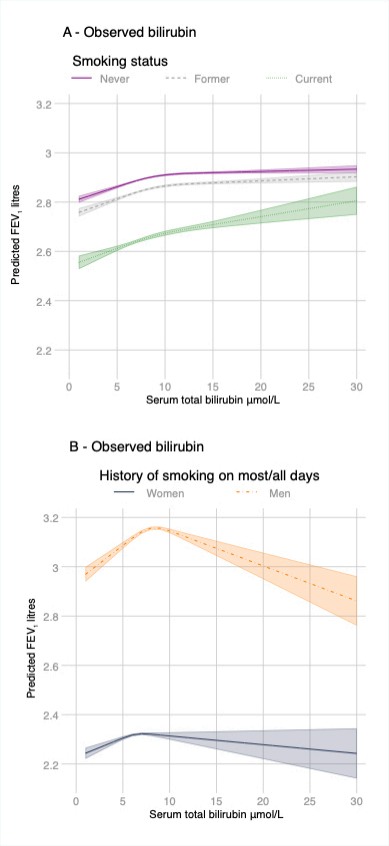
Margins of response by smoking status for FEV_1_ with 95% CIs (shaded) at different levels of bilirubin while holding all other variables at their observed values. Predictions are for age, gender, calendar year, ethnicity (first 40 principal components), height, weight and recruitment centre. Non-linear relationships were captured using cubic spline transformation with three knots placed at the 10th, 50th and 90th percentiles of bilirubin levels.

We found weak cross-sectional relationships between raised serum bilirubin measured at baseline and higher baseline FEV_1_ after adjustment for covariates (table 6, figure 3 and online supplementary figure S1). A three-knot cubic spline transformation was the best fit to the data with a stronger positive relationship at lower values of bilirubin (figure 3). Genetically increased bilirubin was associated with a slightly lower FEV_1_ for most smoking categories (table 6). The highest levels of missing data for FEV_1_ were in smokers and people preferring not to declare smoking status (table 6). Adding inverse probability weights to the regression models to try and account for selection bias reduced the strength of the relationship across most smoking strata (table 6).

**Table 6:** Observational and genetically instrumented relationships between bilirubin and FEV_1_ overall and by smoking status

	Number	Mean FEV_1_ (L)	Missing FEV_1_	Adjusted FEV_1_ (mL) per 5 μmol/L increase in total bilirubin level		FEV_1_ (mL) per 5 μmol/L increase in genetically instrumented bilirubin level‡		FEV_1_ (mL) per 5 μmol/L increase in genetically instrumented bilirubin level with probability weights for missing FEV_1_‡	
				Coefficient (mL) (95% CI)	P value	Coefficient (95% CI)	P value	Coefficient (95% CI)	P value
Overall*	377 294	2.85 (0.78)	91 184 (24%)	21 (19 to 24)	<0.0001	−0.9 (−1.7 to −0.2)	0.025	−0.4 (−1.4 to 0.2)	0.12
Never smokers	205 211	2.88 (0.78)	50 593 (25%)	18 (15 to 21)	<0.0001	−0.6 (−1.6 to 0.3)	0.20	0.3 (−1.3 to 0.7)	0.54
Former smokers	132 709	2.83 (0.76)	30 398 (23%)	22 (18 to 26)	<0.0001	−0.7 (−2.0 to 0.6)	0.28	−0.5 (−1.8 to 0.8)	0.45
Current smokers	38 081	2.77 (0.83)	9800 (26%)	46 (37 to 55)	<0.0001	−3.5 (−7.5 to −0.6)	0.09	−3.1 (−7.0. to 0.6)	0.12
Prefer not to report	1293	2.64 (0.75)	393 (30%)						
Regular smokers†									
Overall	115 247	2.77 (0.78)	26 091 (24%)	36 (32 to 41)	<0.0001	−1.3 (−3.0 to −0.4)	0.13	−1.2 (−2.8 to −0.6)	0.21
Former 1–19	36 606	2.80 (0.76)	8051 (22%)	25 (18 to 33)	<0.0001	0.1 (−2.2 to 2.5)	0.68	0.5 (−2.0 to 3.0)	0.68
Former ≥20	48 296	2.81 (0.77)	11 095 (23%)	33 (26 to 40)	<0.0001	−1.4 (−3.9 to 10.4)	0.26	−1.2 (−3.6 to 11.5)	0.31
Current 1–19	16 032	2.65 (0.80)	4161 (26%)	24 (8 to 40)	0.0003	−4.9 (−11.3 to −14.0)	0.13	−4.7 (−11.3 to −14.2)	0.14
Current ≥20	10 131	2.64 (0.81)	2784 (28%)	2.4 (−18 to 23)	0.82	−5.2 (−14.8 to 4.5)	0.29	−5.3 (−14.8 to 4.6)	0.29

*Adjusted for age, gender, calendar year, ethnicity (first 40 principal components), recruitment centre, height, weight and smoking status.

†Adjusted for pack-years, age, gender, calendar year, ethnicity (first 40 principal components), height, weight, recruitment centre in overall analysis of regular smokers. Participants currently smoking less than one cigarette per day at recruitment are excluded from the smoking subcategories but included in the overall analysis of regular smokers if they had formerly smoked one or more per day and it was possible to calculate pack-years.

‡Adjusted for age, gender, calendar year, ethnicity (first 40 principal components), recruitment centre and smoking status.

Genetically higher bilirubin was associated with slightly lower odds of a family history of lung cancer in smokers and a slightly higher odds of COPD overall (online supplementary table S6). No observational or causal relationships were evident for the negative control cancer outcome (online supplementary table S5). There were non-linear relationships for observed serum total bilirubin with mortality from any cause (online supplementary figure S2) with bilirubin levels below 7 μmol/L associated with substantially raised rates. There was evidence that genetically increased bilirubin was associated with lower all-cause mortality (IRR 0.95 (95% CI 0.91 to 1.0) p=0.034) and cancer mortality (IRR 0.92 (95% CI 0.87 to 0.98); p=0.0075) in participants with a history of smoking regularly (online supplementary table S7). The association with cancer mortality weakened after excluding lung cancer mortality (IRR 0.96 (95% CI 0.92 to 0.99); p=0.027). The remaining sensitivity/supplemental analyses had no meaningful impact on the overall conclusions (supplementary results).

## Discussion

In the present study, we confirm earlier reports of a negative observational relationship between serum total bilirubin and lung cancer that remains after adjustment for smoking pack-years and many other variables. We also report for the first time, that smokers with genetically raised serum bilirubin levels have lower rates of lung cancer and these relationships are strongest in current heavy smokers. These findings are potentially consistent with a second-line antioxidant function where raised serum bilirubin levels are beneficial once the first-line antioxidant defences of the epithelial lining fluid of the lungs are depleted.

### Strengths and limitations

The main advantages of the present study are the large sample size, the use of a powerful and specific genetic instrument, and the longitudinal analysis for lung cancer. The limitations include the short length of follow-up, use of self-report for smoking status and some other variables. Adjustment for variables such as diet, blood counts and cholesterol levels may have further attenuated the observational relationships with our primary and supplementary outcomes. The lowest predicted level for bilirubin was 7 μmol/L and we were unable to determine whether the non-linear relationships seen for observed bilirubin, with much higher disease/mortality rates at very low levels of bilirubin, were potentially causal. The older age at study recruitment and low rates of participation could also be problematic in terms of selection bias. The UK Biobank participants tend to be taller, leaner and there are fewer smokers relative to the UK population.^31^ Rates of smoking-related diseases are therefore reported to be much lower. For example, the prevalence of self-reported COPD in UK Biobank is 0.1% in middle-aged men versus 1% in the Health Survey for England and lung cancer rates are around 50% lower compared with the general population.^31^ Within smokers, heavy smokers are over-represented relative to the general population meaning UK Biobank could be enriched for ‘resistant’ smokers. Selection bias is a recognised problem for UK Biobank and is potentially a problem for our study if people with genetically low bilirubin are less likely to recruited into UK Biobank due to death or poor health.^32^ In contrast to our findings for lung function, two other studies, including a British cohort followed since birth and at lower risk of selection bias, reported improved lung function for people with alleles linked to Gilbert’s syndrome.^29 33^ Considering also the direction of our MR estimates for COPD and a family history of lung cancer, it seems plausible that selection bias could have diluted any relationship between genetically raised bilirubin and improved respiratory outcomes. The increase in the MR-effect estimates for both lung cancer and family history of lung cancer as smoking levels increased is reassuring against chance findings, although some of the smoking specific strata have few events and replicating our results in similarly large cohorts is desirable. Finally, horizontal pleiotropy, whereby another substrate is the real causal agent, is a possibility particularly given the position of *UGT1A1* in a gene complex expressing nine other isoforms with different and overlapping substrates for glucuronidation. Known phenotypes for the low activity SNPs we selected are toxicity to irinotecan chemotherapy (*UGT1A1*) and statin therapy (*SLCO1B1*) but these seem unlikely to explain our results. We can find no other reductions in substrate elimination that could explain the observed relationships with lung cancer.^24^

### Comparison with other studies

Several large cohorts have reported observational relationships between bilirubin and lung cancer. A recent global metabolomic profiling approach in ‘Caucasian’ lung cancer cases and controls identified bilirubin from 403 known metabolites as one of the only consistently significant biomarkers.^30^ The same study validated this finding in a large cohort of 425 660 Taiwanese adults. They reported significant effect modification by smoking status, and for every 0.1 mg/dL (1.71 µmol/L in standard international units) increase of bilirubin, the risks for lung cancer incidence and mortality decreased by 5% and 6% in male smokers, respectively (both p<0.001). There was no association in women possibly due to fewer smokers and cancer events. A similarly large cohort of British adults (n=504 206) reported an 8% decrease for every 0.1 mg/dL (1.71 µmol/L) increase in serum bilirubin levels in men (p<0.001).^11^ The relationship was consistent across genders and no effect modification by smoking was detected (p for interaction >0.05). A third recent study reported a negative relationship with lung cancer with rates 50% lower in the higher serum bilirubin quintile (>11 µmol/L) but this was not statistically significant.^13^ We have now shown that these relationships with serum bilirubin remain in regular smokers after adjusting for additional variables not always accounted for in the earlier analyses including pack-years, passive smoking, occupational smoke exposure and air quality. At this time, we are not aware of any other large-scale prospective studies of *UGT1A1* variation and lung cancer specifically. Several studies have examined the relationship between genetically predicted bilirubin and other clinical outcomes with mixed results that possibly reflects differences in the pathophysiological role for oxidative stress, smoking case-mix, age at recruitment, low power for rare outcomes and study design.^34 35^ Genome-wide association studies have not identified the *UGT1A1* locus in studies of lung cancer, although the number of smokers required to reach the high threshold for genome-wide statistical significance exceeds that available in most cohorts.

### Future directions

Our findings support further studies on the utility of serum bilirubin as a low-cost biomarker for lung cancer risk stratification.^12 20^ Accurate risk stratification is key to the clinical and cost-effectiveness of low-dose CT screening programmes being adopted in the USA and piloted in the UK.^2^ Given the high frequency of lung cancer and the high-cost of false positive results of CT-screening for the patient and provider, even a small improvement in risk prediction could have a meaningful impact and is worth exploring. Further preclinical work could examine whether competitive inhibitors of the UGT1A1 enzyme are legitimate drug targets.

Gilbert’s syndrome is a common condition characterised by moderately elevated levels of unconjugated bilirubin and intermittent episodes of jaundice. Ten per cent of people with European or East Asian ancestry and 25% of people with equatorial African descent have at least two functional variants of *UGT1A1* associated with the condition, although not all will meet the bilirubin threshold for diagnosing Gilbert’s syndrome.^3 36^ The high frequency of different *UGT1A1* variants causing mild hereditary hyperbilirubinaemia has led to speculation of balancing natural selection whereby physiological benefits of hyperbilirubinaemia are countered by the neurotoxic impact on infants. Under this scenario, functional alleles reach a high frequency but never fixate.^3^ Our results suggest that for UK Biobank participants self-reporting as current smokers and with a phenotype consistent with Gilbert’s syndrome (serum total bilirubin >17 μmol/L), rates of lung cancer are 35%–50% lower and mortality 20%–40% lower than a similar group of smokers with bilirubin levels <5 μmol/L. Protection against smoke oxidants could have been advantageous for early humans once fire was discovered and widely used for lighting, warmth, cooking in poorly ventilated caves or dwellings.^37^ However, protection against infectious diseases is strongly implicated as the primary driver of existing examples of balancing selection and raised serum bilirubin does seem to inhibit contagious agents including group B streptococcus,^38^ hepatitis C^39^ and malaria parasites.^40^ As more events accrue in UK Biobank and the data become fully linked with primary healthcare records, it will be feasible to investigate a role for serum bilirubin in other infectious and non-communicable diseases.

### Summary

We have shown that adult smokers in UK Biobank with genetically raised bilirubin have lower rates of lung cancer, supporting endogenous antioxidant properties. Further work is required to establish whether bilirubin is a useful low-cost biomarker for improving risk prediction and a legitimate therapeutic target for disease prevention.

## References

[1] Uk CR Worldwide cancer statistics, 2015 Available: https://www.cancerresearchuk.org/health-professional/cancer-statistics/worldwide-cancer [Accessed 13 May 2015].

[2] Raymakers AJN, Mayo J, Lam S, et al Cost-effectiveness analyses of lung cancer screening strategies using low-dose computed tomography: a systematic review. Appl Health Econ Health Policy 2016;14:409–18. 10.1007/s40258-016-0226-5 26873091

[3] Beutler E, Gelbart T, Demina A Racial variability in the UDP-glucuronosyltransferase 1 (UGT1A1) promoter: a balanced polymorphism for regulation of bilirubin metabolism? Proc Natl Acad Sci U S A 1998;95:8170–4. 10.1073/pnas.95.14.8170 9653159 PMC20948

[4] Premawardhena A, Fisher CA, Liu YT, et al The global distribution of length polymorphisms of the promoters of the glucuronosyltransferase 1 gene (UGT1A1): hematologic and evolutionary implications. Blood Cells Mol Dis 2003;31:98–101. 10.1016/S1079-9796(03)00071-8 12850492

[5] Chen W, Maghzal GJ, Ayer A, et al Absence of the biliverdin reductase-a gene is associated with increased endogenous oxidative stress. Free Radic Biol Med 2018;115:156–65. 10.1016/j.freeradbiomed.2017.11.020 29195835

[6] Dennery PA, McDonagh AF, Spitz DR, et al Hyperbilirubinemia results in reduced oxidative injury in neonatal Gunn rats exposed to hyperoxia. Free Radic Biol Med 1995;19:395–404. 10.1016/0891-5849(95)00032-S 7590389

[7] Nakagami T, Toyomura K, Kinoshita T, et al A beneficial role of bile pigments as an endogenous tissue protector: anti-complement effects of biliverdin and conjugated bilirubin. Biochim Biophys Acta 1993;1158:189–93. 10.1016/0304-4165(93)90013-X 8399320

[8] Stocker R, Yamamoto Y, McDonagh AF, et al Bilirubin is an antioxidant of possible physiological importance. Science 1987;235:1043–6. 10.1126/science.3029864 3029864

[9] Uhlén M, Fagerberg L, Hallström BM, et al Proteomics. tissue-based map of the human proteome. Science 2015;347:1260419. 10.1126/science.1260419 25613900

[10] Titz B, Szostak J, Sewer A, et al Multi-omics systems toxicology study of mouse lung assessing the effects of aerosols from two heat-not-burn tobacco products and cigarette smoke. Comput Struct Biotechnol J 2020;18:1056–73. 10.1016/j.csbj.2020.04.011 32419906 PMC7218232

[11] Horsfall LJ, Rait G, Walters K, et al Serum bilirubin and risk of respiratory disease and death. JAMA 2011;305:691–7. 10.1001/jama.2011.124 21325185

[12] Lim J-E, Kimm H, Jee SH Combined effects of smoking and bilirubin levels on the risk of lung cancer in Korea: the severance cohort study. PLoS One 2014;9:e103972. 10.1371/journal.pone.0103972 25100210 PMC4123988

[13] Kühn T, Sookthai D, Graf ME, et al Albumin, bilirubin, uric acid and cancer risk: results from a prospective population-based study. Br J Cancer 2017;117:1572–9. 10.1038/bjc.2017.313 28898231 PMC5680462

[14] Wu X, Wen CP, Ye Y, et al Personalized risk assessment in never, light, and heavy smokers in a prospective cohort in Taiwan. Sci Rep 2016;6:36482. 10.1038/srep36482 27805040 PMC5090352

[15] Van Hoydonck PG, Temme EH, Schouten EG Serum bilirubin concentration in a Belgian population: the association with smoking status and type of cigarettes. Int J Epidemiol 2001;30:1465–72. 10.1093/ije/30.6.1465 11821365

[16] Haj Mouhamed D, Ezzaher A, Neffati F, et al Effect of cigarette smoking on plasma uric acid concentrations. Environ Health Prev Med 2011;16:307–12. 10.1007/s12199-010-0198-2 21431788 PMC3156839

[17] Burgess S, Thompson SG, Burgess S Mendelian randomization: methods for using genetic variants in causal estimation. Boca Raton, FL: CRC Press, Taylor & Francis Group, 2015.

[18] Sudlow C, Gallacher J, Allen N, et al UK biobank: an open access resource for identifying the causes of a wide range of complex diseases of middle and old age. PLoS Med 2015;12:e1001779. 10.1371/journal.pmed.1001779 25826379 PMC4380465

[19] Hanscombe KB, Coleman JRI, Traylor M, et al ukbtools: an R package to manage and query UK Biobank data. PLoS One 2019;14:e0214311. 10.1371/journal.pone.0214311 31150407 PMC6544205

[20] Bycroft C, Freeman C, Petkova D, et al Genome-wide genetic data on ~500,000 UK Biobank participants. bioRxiv 2017;166298.

[21] Kang T-W, Kim H-J, Ju H, et al Genome-wide association of serum bilirubin levels in Korean population. Hum Mol Genet 2010;19:3672–8. 10.1093/hmg/ddq281 20639394 PMC2928134

[22] Johnson AD, Kavousi M, Smith AV, et al Genome-wide association meta-analysis for total serum bilirubin levels. Hum Mol Genet 2009;18:2700–10. 10.1093/hmg/ddp202 19414484 PMC2701336

[23] Chen G, Ramos E, Adeyemo A, et al UGT1A1 is a major locus influencing bilirubin levels in African Americans. Eur J Hum Genet 2012;20:463–8. 10.1038/ejhg.2011.206 22085899 PMC3306855

[24] Shin S-Y, Fauman EB, Petersen A-K, et al An atlas of genetic influences on human blood metabolites. Nat Genet 2014;46:543–50. 10.1038/ng.2982 24816252 PMC4064254

[25] Namjou B, Marsolo K, Lingren T, et al A GWAS study on liver function test using eMERGE network participants. PLoS One 2015;10:e0138677. 10.1371/journal.pone.0138677 26413716 PMC4586138

[26] Bielinski SJ, Chai HS, Pathak J, et al Mayo genome consortia: a genotype-phenotype resource for genome-wide association studies with an application to the analysis of circulating bilirubin levels. Mayo Clin Proc 2011;86:606–14. 10.4065/mcp.2011.0178 21646302 PMC3127556

[27] Brown KF, Rumgay H, Dunlop C, et al The fraction of cancer attributable to modifiable risk factors in England, Wales, Scotland, Northern Ireland, and the United Kingdom in 2015. Br J Cancer 2018;118:1130–41. 10.1038/s41416-018-0029-6 29567982 PMC5931106

[28] Burgess S, Davey Smith G, Davies N Guidelines for performing mendelian randomization investigations. Wellcome Open Research, 2019. 10.12688/wellcomeopenres.15555.1 PMC7384151 32760811

[29] Horsfall LJ, Hardy R, Wong A, et al Genetic variation underlying common hereditary hyperbilirubinaemia (Gilbert's syndrome) and respiratory health in the 1946 British birth cohort. J Hepatol 2014;61:1344–51. 10.1016/j.jhep.2014.07.028 25086287

[30] Wen C-P, Zhang F, Liang D, et al The ability of bilirubin in identifying smokers with higher risk of lung cancer: a large cohort study in conjunction with global metabolomic profiling. Clin Cancer Res 2015;21:193–200. 10.1158/1078-0432.CCR-14-0748 25336700 PMC4286447

[31] Fry A, Littlejohns TJ, Sudlow C, et al Comparison of sociodemographic and health-related characteristics of UK Biobank participants with those of the general population. Am J Epidemiol 2017;186:1026–34. 10.1093/aje/kwx246 28641372 PMC5860371

[32] Munafò MR, Tilling K, Taylor AE, et al Collider scope: when selection bias can substantially influence observed associations. Int J Epidemiol 2018;47:226–35. 10.1093/ije/dyx206 29040562 PMC5837306

[33] Curjuric I, Imboden M, Adam M, et al Serum bilirubin is associated with lung function in a Swiss general population sample. Eur Respir J 2014;43:1278–88. 10.1183/09031936.00055813 24177000

[34] Vitek L, Hubacek JA, Pajak A, et al Association between plasma bilirubin and mortality. Ann Hepatol 2019;18:379–85. 10.1016/j.aohep.2019.02.001 31054979

[35] Stender S, Frikke-Schmidt R, Nordestgaard BG, et al Genetically elevated bilirubin and risk of ischaemic heart disease: three Mendelian randomization studies and a meta-analysis. J Intern Med 2013;273:59–68. 10.1111/j.1365-2796.2012.02576.x 22805420

[36] Horsfall LJ, Zeitlyn D, Tarekegn A, et al Prevalence of clinically relevant UGT1A alleles and haplotypes in African populations. Ann Hum Genet 2011;75:236–46. 10.1111/j.1469-1809.2010.00638.x 21309756

[37] Hubbard TD, Murray IA, Bisson WH, et al Divergent Ah receptor ligand selectivity during hominin evolution. Mol Biol Evol 2016;33:2648–58. 10.1093/molbev/msw143 27486223 PMC5026259

[38] Hansen R, Gibson S, De Paiva Alves E, et al Adaptive response of neonatal sepsis-derived group B Streptococcus to bilirubin. Sci Rep 2018;8:6470. 10.1038/s41598-018-24811-3 29691444 PMC5915570

[39] Zhu Z, Wilson AT, Luxon BA, et al Biliverdin inhibits hepatitis C virus nonstructural 3/4A protease activity: mechanism for the antiviral effects of heme oxygenase? Hepatology 2010;52:1897–905. 10.1002/hep.23921 21105106 PMC3058505

[40] Kumar S, Guha M, Choubey V, et al Bilirubin inhibits Plasmodium falciparum growth through the generation of reactive oxygen species. Free Radic Biol Med 2008;44:602–13. 10.1016/j.freeradbiomed.2007.10.057 18070610

