# Supplementary material for: Genetically raised serum bilirubin levels and lung cancer: a cohort study and Mendelian randomisation using UK Biobank
Source: Thorax. 2020 Aug 27;75(11):955–64. doi: 10.1136/thoraxjnl-2020-214756 (PMC7569373; doi:10.1136/thoraxjnl-2020-214756)
Supplement: Supplementary data [file thoraxjnl-2020-214756supp001.pdf]

Supplementary methods

Data source

Participants attended 22 centres with locations selected to ensure representation of people from different socioeconomic, ethnic and urban-rural backgrounds. This ongoing study collects data from questionnaires, sample assays, physical measures, genome-wide genotyping and follow-up for a wide range of health-related outcomes some of which are linked to national registers and electronic health records. Genome-wide genotype data was available for two microarrays; the Affymetrix UK Biobank Axiom® array for most participants and the Applied Biosystems™ UK BiLEVE Axiom™ Array by Affymetrix for a smaller subset (n=49,950)[1]. Details on the quality control and imputation of SNPs, indels and structural variants are reported elsewhere[1].

Observational associations – further details

For bilirubin and the time scale (age), we explored non-linear relationships by applying cubic spline-interpolation using Harrell's default percentiles and selecting the transformation that minimised the Akaike and Bayesian information criteria (AIC/BIC)[2]. We applied a user-written programme for data visualisation[3]. Serum bilirubin data is slightly right-skewed, and we also checked for non-linear relationships following log-transformation. For both the observed and genetically predicted bilirubin levels, we checked for proportionality of associations with age by testing interaction terms. All continuous covariates were parameterised as linear in the regression models and Wald tests were used for calculating p-values for categorical variables and spline transformations.

Genetically instrumented associations – further details

We combined the effects of the two SNPs on bilirubin levels to estimate the incident rate ratios (IRRs) for lung cancer per five µmol/L increase genetically predicted bilirubin using one-sample MR and the two-stage predictor substitution (2SPS) method[4]. In brief, bilirubin levels were regressed against the two SNPs to give the fitted "unconfounded" bilirubin levels. We modelled the SNPs as three-level categories to capture non-additive relationships with serum bilirubin. These fitted values were then used as the exposure in a Poisson model of lung cancer incidence. Robust standard errors were calculated to account for the added uncertainty of using previously fitted values as the exposure in the second stage of the regression[4].

Horsfall LJ, *et al. Thorax* 2020; 75:955–964. doi: 10.1136/thoraxjnl-2020-214756

We examined whether other factors associated with lung cancer (FEV_1/COPD/emphysema and family history of lung cancer) were intervening/mediating variables in the relationship between bilirubin and lung cancer. The method of spirometry at baseline is reported in detail elsewhere[5] and we used the maximum value of the measures meeting the assessor's acceptability criteria. We estimated the observational relationship between bilirubin and baseline FEV_1 using linear regression. We identified and excluded outlier values of bilirubin and FEV_1 using multivariate approach (blocked adaptive computationally efficient outlier nominators algorithm) with a 15% threshold of the chi-squared distribution used to separate outliers from non-outliers[6].

We used a similar approach, the two stage least squares method (2SLS), to estimate the causal cross-sectional relationship between bilirubin and FEV_1[4]. FEV_1 was missing for approximately 25% of participants and were missing not at random with respect to other risk factors. We used inverse probability weighting in an attempt to reduce the impact of any selection bias where each participant was weighted by their likelihood of providing an acceptable FEV_1 reading. Probability weights were calculated using a logistic regression where missing FEV_1 was the outcome and covariates included age, gender, height, weight, smoking status, lung cancer events, genotypes and bilirubin levels. Due even higher levels of missing FEV_1 data of around 50% for smokers once applying the ERS/ATS criteria for FEV_1 reproducibility, this analysis was not done.

Recent use of respiratory medication was self-reported by participants at baseline and included treatments for asthma, hay fever, emphysema, chronic bronchitis, COPD, cystic fibrosis, alpha-1 antitrypsin deficiency, sarcoidosis, bronchiectasis, idiopathic pulmonary fibrosis, fibrosing alveolitis/unspecified alveolitis, silicosis, asbestosis and tuberculosis. These medications could affect FEV_1 readings and so we assessed the impact of excluding participants reporting to be on these drugs.

Interactions with other variables

Other environmental sources of oxidants include passive smoking at home or in the workplace and air pollution. As a supplemental analysis, we examined whether there were interactions between these variables, serum bilirubin and lung cancer risk. Only participants who reported to not smoke regularly had data available on smoking outside of the home.

Negative control

We included a composite negative control outcome of neurological, haematological cancers and melanomas. Smoke exposure has a lower aetiological role in these cancers[7] and we would therefore weak to no relationship across smoking strata if serum bilirubin is functioning as an endogenous antioxidant. We used a composite outcome to ensure there were adequate numbers of events in the smaller smoking sub-categories. The ICD9/10 codes used to define the negative control cancer outcome are in the table below:

ICD10	ICD9
C43	172
C70	173
C71	191
C72	200
C81	201
C82	202
C83	203
C84	204
C85	205
C86	206
C88	207
C91	208
C92	
C93	
C94	
C95	
C96	

Other sensitivity/supplemental analyses

Other potential confounding variables for the observational relationships with bilirubin included passive smoking, occupational exposure to smoke, antioxidant supplements (vitamin C, vitamin E and β-carotene), social deprivation, air pollution (NO_2 and PM2.5), and liver blood tests (alkaline phosphatase, alanine aminotransferase and gamma glutamyl transferase). We examined the effect of adjustment for these additional variables for a sub-sample with complete data on all covariates. We also adjusted for the microarray identity (UK BiLEVE) under the caveat that this could introduce collider bias for respiratory outcomes.

Unconjugated bilirubin is the specific endogenous substrate for the UGT1A1 enzyme. Direct/conjugated bilirubin was recorded for a subset of participants (n=306,070), which means by subtraction (serum total bilirubin minus direct bilirubin=indirect bilirubin) we could

also estimate the causal relationships indirect/unconjugated bilirubin. These estimates could be more precise than using total serum bilirubin, which will also capture increases the conjugated fraction due to common diseases.

Serum total bilirubin has been associated with a range of other age-related diseases. We therefore ran supplemental analyses with mortality from any cause and cancer mortality as the outcomes under the caveat that we expected weaker associations due to the inclusion of events unrelated to oxidant exposure. Complete mortality data was available up to 31st January 2018 for England and Wales and 30th October 2016 for Scotland.

Finally, we checked the effect of restricting the MR analyses to the *UGT1A1* rs887829 variant.

Supplementary results

Including additional covariates (occupational smoke exposure, household smoke exposure, antioxidant supplements, waist circumference, air pollution – NO_2 and PM2.5, liver enzymes) also had no meaningful impact on the observational or causal estimates for any outcomes (Figure S1). Excluding participants on respiratory medication from the analyses of FEV_1 had no impact on the estimates. We found no relationships between observed or genetically predicted bilirubin and the negative control cancers though incident rates by smoking status need to be interpreted with caution because smokers may die from lung cancer before they can develop these cancers (Table S4). We reran the analyses using unconjugated bilirubin instead of total bilirubin but this did not improve the precision of causal estimates (Table S5). Excluding rs4149056 from the MR analysis had a no real impact and changed the IRRs by >= 0.01. There was no strong evidence of interaction with other variables that influence exposure to oxidants, though these variables had a much weaker effect on lung cancer relative to cigarette smoking. Genetically raised bilirubin was weakly associated with lower rates of lung cancer in first degree relatives of smokers and slightly higher prevalence of self-reported COPD/emphysema at baseline (Table S6).

We found non-linear relationships (four-knot cubic spline transformation) between serum total bilirubin levels and mortality from any cause with much higher rates at very low bilirubin levels (Figure S2). Unlike for lung cancer there was limited evidence of a multiplicative interactions with smoking status and low bilirubin was associated with excess mortality for never and former smokers. For participants with a history of regular smoking, the associations were also non-linear and broadly similar for women and men (Figure S2).

Horsfall LJ, *et al. Thorax* 2020; 75:955–964. doi: 10.1136/thoraxjnl-2020-214756

There was an uptick in rates at higher levels of bilirubin for current smokers (Figure S2 A & C) but no such trend was apparent after adjusting for smoking intensity and duration (pack-years) (Figure S2 B & D). There were negative associations with genetically predicted serum total bilirubin levels and mortality that were stronger in regular smokers (Table S7). There was also an association with cancer mortality in participants with a history of smoking regularly that remained after excluding lung cancer deaths from the analysis (IRR:0.96 (95%CI: 0.92,0.99);p=0.027) suggesting a role for bilirubin in other cancers related to smoking (data not shown).

Horsfall LJ, *et al. Thorax* 2020; 75:955–964. doi: 10.1136/thoraxjnl-2020-214756

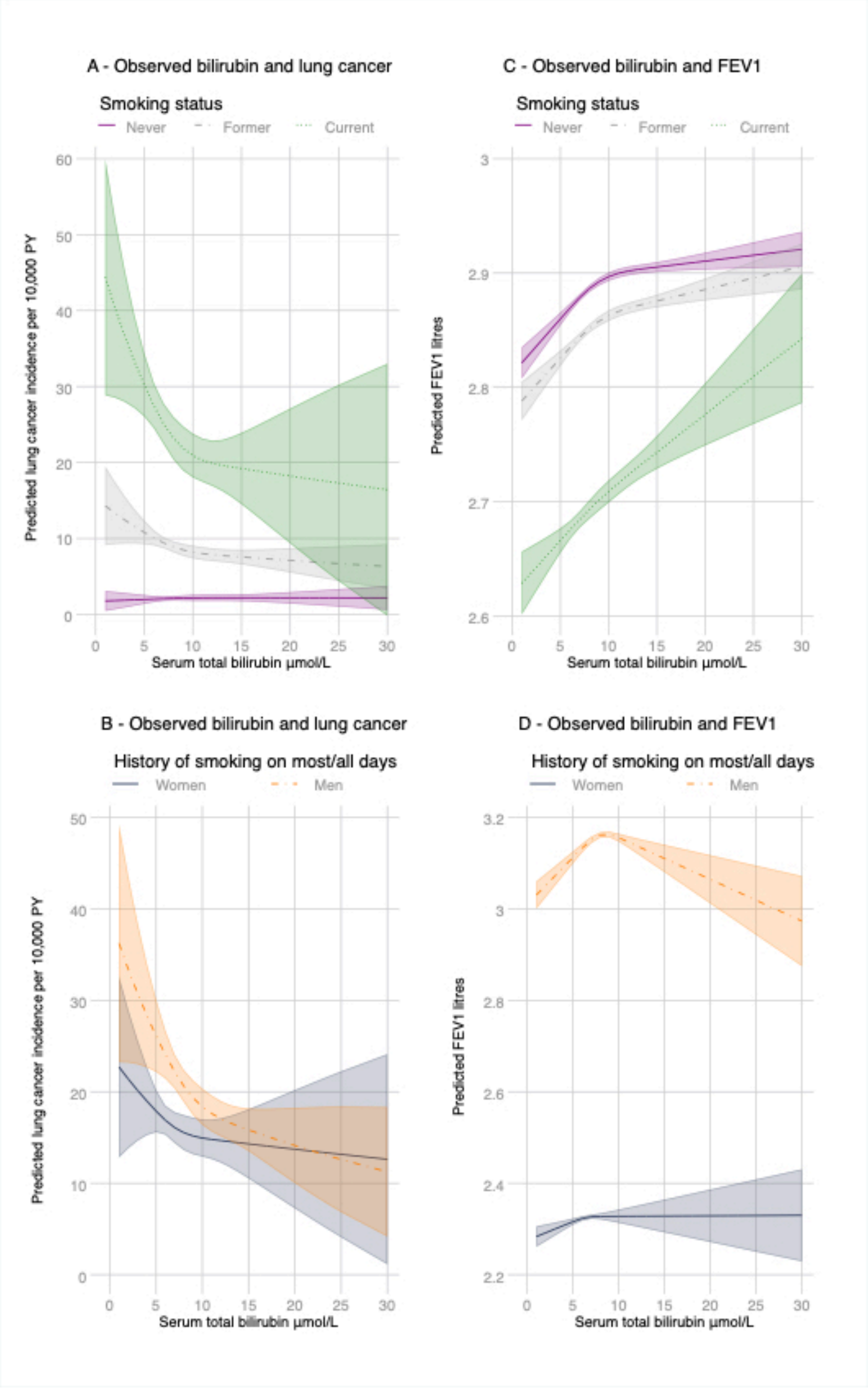

Figure S1: Adjusted associations of observed serum bilirubin with lung cancer (A and B) and FEV_1 (C and D) showing the predictive margins (with other variables held at their observed levels and 95%Cis shaded) across smoking status (top panel A and C) and for participants with a history of regularly smoking at least one cigarette per day (bottom panel B and D). Predictions account for age, gender, calendar year, ethnicity (first 40 principal components), height, weight, waist circumference, recruitment centre, passive smoking, occupational exposure to smoke, antioxidant supplements (vitamin C, vitamin E and β-carotene), social deprivation (Townsend score), air pollution, and liver blood tests (alkaline phosphatase, alanine aminotransferase and gamma glutamyl transferase). Non-linear associations were captured using cubic spline transformation with three knots placed at the 10th, 50th and 90th percentiles of bilirubin levels.

6

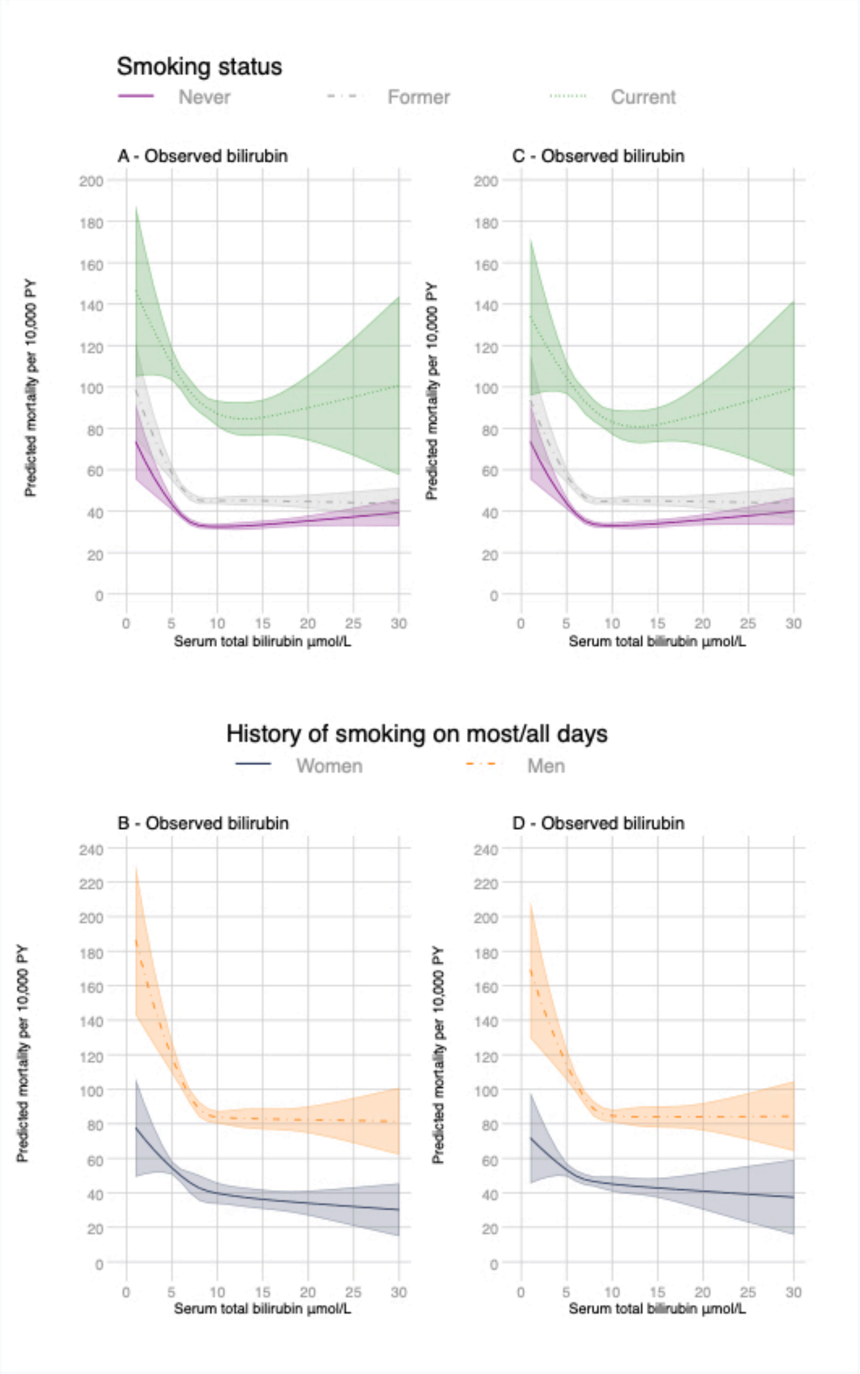

Figure S2: Adjusted associations of observed serum bilirubin with mortality from any cause showing the predictive margins (with other variables held at their observed levels and 95%Cis shaded) across smoking status (top panels A and C) and for participants with a history of regularly smoking at least one cigarette per day (bottom panel B and D). Predictions account for age, gender, calendar year, ethnicity (first 40 principal components), height, weight, recruitment centre, diastolic/systolic bold pressure (Panels A and B) and further adjusted for passive smoking, occupational exposure to smoke, waist circumference, antioxidant supplements (vitamin C, vitamin E and β-carotene), social deprivation (Townsend score), air pollution, and liver blood tests (alkaline phosphatase, alanine aminotransferase and gamma glutamyl transferase). Non-linear associations were captured using cubic spline transformation with four knots placed at the 5th, 35th, 65th and 95th percentiles of bilirubin level.

Horsfall LJ, *et al. Thorax* 2020; 75:955–964. doi: 10.1136/thoraxjnl-2020-214756

Table S1: The observational and genetically instrumented relationships reported as incidence rate ratios (IRRs) between bilirubin and lung cancer overall and by smoking status before and after adjusting for covariates.

	Observational				*Genetically instrumented*			
	Unadjusted IRR *per 5 μmol/L increase* (95%CI)	p-value	Adjusted IRR *per 5 μmol/L increase* (95%CI)*	p-value	Unadjusted IRR *per 5 μmol/L increase* (95%CI)	p-value	Adjusted IRR *per 5 μmol/L increase* (95%CI)**	p-value
Overall	0.79 (0.74,0.84)	<0.0001	0.85 (0.80,0.92)	<0.0001	0.91 (0.83,0.99)	0.024	0.90 (0.83,0.99)	0.023
Never smokers	1.01 (0.88,1.15)	0.91	1.00 (0.87,1.15)	0.97	0.98 (0.78,1.22)	0.84	0.98 (0.78,1.22)	0.87
Former smokers	0.91 (0.84,0.99)	0.034	0.87 (0.79,0.96)	0.0037	0.95 (0.84,1.07)	0.40	0.95 (0.84,1.08)	0.40
Current smokers	0.78 (0.69,0.89)	<0.0001	0.74 (0.63,0.86)	<0.0001	0.83 (0.72,0.96)	0.013	0.83 (0.72,0.96)	0.011
Prefer not to report								
Regular smokers								
Overall	0.81 (0.76,0.88)	<0.0001	0.77 (0.70,0.84)	<0.0001	0.89 (0.81,0.99)	0.030	0.89 (0.80,0.99)	0.028
Former 1-19	0.97 (0.81,1.15)	0.069	0.95 (0.80,1.12)	0.52	1.05 (0.81,1.35)	0.73	1.04 (0.81,1.33)	0.74
Former ≥20	0.84 (0.75,0.94)	0.005	0.79 (0.69,0.90)	0.0006	0.91 (0.77,1.07)	0.26	0.92 (0.79,1.08)	0.29
Current 1-19	0.92 (0.76,1.12)	0.45	0.88 (0.70,1.13)	0.32	0.94 (0.76,1.17)	0.57	0.94 (0.76,1.17)	0.57
Current ≥20	0.82 (0.67, 1.00)	0.071	0.76 (0.60,0.95)	0.021	0.73 (0.59,0.91)	0.0054	0.72 (0.58,0.90)	0.0037

*Age, gender, calendar year, ethnicity (first 40 principal components), height, weight, recruitment centre and smoking status (pack-years in overall analysis of regular smokers).

** Age, gender, calendar year, ethnicity (first 40 principal components), recruitment centre and smoking status

Horsfall LJ, *et al. Thorax* 2020; 75:955–964. doi: 10.1136/thoraxjnl-2020-214756

Table S2: Baseline characteristics of UK Biobank participants by UGT1A1 rs887829 genotype. All continuous variables are mean values with ± 1 standard or medians for skewed data if interquartile ranges (IQRs) are specified.

	UGT1A1 rs887829 genotype					
	Total	CC	CT	TT		
	N=377,294	N=177,209	N=162,815	N=37,270	p-value**	Test
Sex	174,881 (46.4%)	82,146 (46.4%)	75,470 (46.4%)	17,265 (46.3%)	0.99	Pearson's chi-squared
Age at recruitment (IQR)	58.9 (51.4-64.0)	58.9 (51.4-63.9)	58.9 (51.3-64.0)	59.0 (51.4-64.0)	0.32	Kruskal-Wallis
Weight (kg)	78.3 (15.9)	78.3 (15.9)	78.3 (15.9)	78.4 (15.9)	0.75	ANOVA
Height (cm)	168.8 (9.2)	168.8 (9.2)	168.8 (9.2)	168.9 (9.2)	0.22	ANOVA
Waist circumference	90.4 (13.5)	90.4 (13.5)	90.4 (13.5)	90.4 (13.4)	0.83	ANOVA
BMI	27.4 (4.8)	27.4 (4.7)	27.4 (4.8)	27.4 (4.8)	0.97	ANOVA
Smoking status					0.35	Pearson's chi-squared
Never	205,211 (54.4%)	96,535 (54.5%)	88,303 (54.2%)	20,373 (54.7%)		
Former	132,709 (35.2%)	62,125 (35.1%)	57,566 (35.4%)	13,018 (34.9%)		
Current	38,081 (10.1%)	17,926 (10.1%)	16,389 (10.1%)	3,766 (10.1%)		
Missing	1,293 (0.3%)	623 (0.4%)	557 (0.3%)	113 (0.3%)		
Pack years of smoking (IQR)*	19.5 (10.0-32.5)	19.5 (10.0-32.6)	19.5 (10.0-32.5)	19.4 (10.1-32.2)	0.25	Kruskal-Wallis
Occupational smoke exposure	99,914 (26.5%)	47,031 (26.5%)	42,908 (26.4%)	9,975 (26.8%)	0.20	Pearson's chi-squared
Exposure to smoke at home	34,374 (9.1%)	16,141 (9.1%)	14,873 (9.1%)	3,360 (9.0%)	0.77	Pearson's chi-squared
Antioxidant supplements	102,430 (27.1%)	48,194 (27.2%)	44,100 (27.1%)	10,136 (27.2%)	0.75	Pearson's chi-squared
Nitrogen dioxide air pollution µg/cubic metre (IQR); 2010	25.55 (20.98-30.39)	25.57 (20.99-30.41)	25.53 (20.98-30.38)	25.55 (20.94-30.36)	0.68	Kruskal-Wallis
Particulate matter air pollution µg/cubic metre (pm2.5) (IQR); 2010	9.88 (9.23-10.49)	9.88 (9.23-10.49)	9.88 (9.23-10.49)	9.87 (9.22-10.49)	0.50	Kruskal-Wallis
Townsend deprivation index (IQR)	-2.4 (-3.7-0.1)	-2.3 (-3.7-0.1)	-2.4 (-3.7-0.0)	-2.4 (-3.7-0.1)	0.39	Kruskal-Wallis

IQR=Interquartile range

**Previously calculated for 109,312 participants reporting to regularly smoke at least one cigarette/day and who also reported smoking duration.*

***Univariable association with genotype*

Table S3: The predicted margins (incidence rate) for lung cancer across mid-points of serum bilirubin quintiles for observational (non-linear) and genetically instrumented associations between serum bilirubin and lung cancer by smoking status.

	Serum bilirubin value µmol/L *	4	7	8	10	17	31
Observational predicted incidence rate per 10,000 PYs (95%CI)**	Never	2.4 (1.5,3.2)	2.1 (1.8,2.3)	2.1 (1.7,2.3)	1.9 (1.6,2.3)	2.1 (1.6,2.5)	2.4 (0.9,3.9)
	Former	11.8 (9.5,14)	9.4 (8.7,10.1)	8.8 (8.1,9.5)	8.1 (7.3,8.9)	7.3 (6.2,8.5)	6.4 (3.5,9.3)
	Current	48.8 (41,56.6)	35.1 (32.1,38)	31.9 (28.7,35.1)	28.0 (24.7,31.3)	23.2 (15.6,30.9)	17.6 (0.1,35)
Genetically instrumented incidence rate per 10,000 PYs (95%CI)***	Never	2.1 (1.5,2.6)	2.0 (1.7,2.4)	2.0 (1.8,2.3)	2.0 (1.8,2.3)	2 (1.2,2.7)	1.9 (0.1,3.6)
	Former	9.2 (7.9,10.6)	9.0 (8.2,9.7)	8.9 (8.2,9.5)	8.7 (8.1,9.3)	8 (6.3,9.7)	7.0 (3.3,10.7)
	Current	41.8 (35.3,48.4)	37.4 (34.1,40.8)	36.1 (33.4,38.8)	33.5 (30.9,36.1)	25.9 (19.6,32.2)	16.0 (6.2,25.7)
Observational predicted incidence rate per 10,000 PYs (95%CI)**	Former 1-19	6.4 (3.5,9.4)	7.6 (6.4,8.8)	7.9 (6.5,9.2)	7.9 (6.3,9.6)	6.3 (4.1,8.6)	4 (-0.3,8.3)
	Former ≥20	25.1 (19.3,30.9)	17.5 (15.9,19.1)	15.9 (14.2,17.5)	14 (12.3,15.7)	12 (9.5,14.6)	9.6 (3.7,15.5)
	Current 1-19	41.2 (30.2,52.2)	34.5 (29.5,39.4)	32.9 (27.3,38.6)	31.3 (25.3,37.2)	30.7 (13.2,48.1)	30.5 (-21.4,82.4)
	Current ≥20	91 (69.7,112.2)	68 (59.1,76.9)	62.9 (53,72.8)	57 (47.1,66.9)	50.8 (18.7,83)	42.9 (-36.5,122.3)
Genetically instrumented incidence rate per 10,000 PYs (95%CI)***	Former 1-19	7.1 (5,9.3)	7.3 (6,8.6)	7.4 (6.3,8.5)	7.5 (6.4,8.6)	8 (4.6,11.3)	8.9 (-0.5,18.3)
	Former ≥20	17.2 (14,20.3)	16.3 (14.6,18)	16 (14.6,17.4)	15.5 (14.1,16.8)	13.7 (9.9,17.4)	10.9 (3.4,18.4)
	Current 1-19	37.5 (28.3,46.7)	36.1 (30.9,41.4)	35.7 (31.3,40.1)	34.8 (30.5,39.1)	31.9 (20.3,43.5)	27.2 (2.4,51.9)
	Current ≥20	97.1 (74.3,120)	79.7 (69.4,90.1)	74.7 (66.5,82.9)	65.5 (57.7,73.2)	41.3 (25.6,57.1)	17.6 (0.9,34.3)

*Mid-point value of serum bilirubin quintiles plus 17 µmol/L for assessing the rates above the bilirubin level often used to diagnose Gilbert's syndrome.

**Holding age, gender, calendar year, height, weight, ethnicity (first 40 principal components) and recruitment centre at observed values for the full dataset.

***Holding age, gender, calendar year, ethnicity (first 40 principal components) and recruitment centre at observed values for the full dataset.

Table S4: The observational and genetically instrumented relationships between bilirubin and negative control cancers (neurological, haematological and melanomas) overall and by smoking status.

			*Adjusted observational association**		*Genetically instrumented estimate including covariates**	
	Events	Rate	IRR *per 5 µmol/L increase* (95%CI)	p-value	IRR *per 5 µmol/L increase* (95%CI)	p-value
Overall	4448	17.3 (16.8,17.9)	0.99 (0.95,1.02)	0.50	0.98 (0.93,1.03)	0.45
Never smokers	2311	16.5 (15.8,17.2)	1.01 (0.87,1.15)	0.77	1.01 (0.94,1.09)	0.73
Former smokers	1734	19.3 (18.4,20.3)	0.97 (0.91,1.03)	0.27	0.95 (0.87,1.04)	0.26
Current smokers	382	14.7 (13.3,16.3)	0.97 (0.83,1.13)	0.63	0.92 (0.76,1.13)	0.45
Regular smokers						
Overall*	1344	17.9 (16.9,18.8)	1.00 (0.93,1.07)	0.92	0.98 (0.88,1.08)	0.64
Former 1-19	422	17.0 (15.4,18.7)	0.96 (0.85,1.09)	0.54	1.08 (0.91,1.28)	0.41
Former ≥20	674	20.7 (19.2,22.3)	1.02 (0.95,1.11)	0.74	0.92 (0.79,1.06)	0.24
Current 1-19	146	13.3 (11.3,15.7)	1.09 (0.85,1.40)	0.50	1.00 (0.74,1.34)	0.97
Current ≥20	102	14.8 (12.2,18.0)	0.88 (0.61,1.28)	0.46	0.97 (0.67,1.40)	0.98

*Age, gender, calendar year, ethnicity (first 40 principal components) recruitment centre and smoking status. Estimates derived using a one-sample MR approach and the two-stage predictor substitution (2SPS) method.
**Adjusted for pack-years in overall analysis of regular smokers. Participants currently smoking less than 1 cigarette per day at recruitment are excluded from the smoking sub-categories but included in the overall analysis of regular smokers if they had formerly smoked one or more per day and it was possible to calculate pack-years.

Horsfall LJ, *et al. Thorax* 2020; 75:955–964. doi: 10.1136/thoraxjnl-2020-214756

Table S5: Genetically instrumented relationships between unconjugated bilirubin (serum total bilirubin minus direct bilirubin) and lung cancer overall and by smoking status.

	IRR per 5 μmol/L increase (95%CI)*		Predicted incidence change in 10,000PYs per 5 μmol/L increase (95%CI)*
Overall	0.88 (0.79,0.98)	0.023	-0.95 (-1.77,-0.13)
Never smokers	0.98 (0.74,1.29)	0.86	-0.05 (-0.61,0.51)
Former smokers	0.94 (0.80,1.10)	0.41	-0.58 (-1.95,0.8)
Current smokers	0.79 (0.66,0.95)	0.011	-8.1 (-14.35,-1.84)
Regular smokers			
*Overall***	0.89 (0.80,0.99)	0.032	-2.08 (-4,-0.16)
Former 1-19	1.05 (0.77,1.44)	0.76	0.36 (-1.99,2.71)
Former ≥20	0.90 (0.73,1.10)	0.29	-1.72 (-4.91,1.47)
Current 1-19	0.93 (0.71,1.21)	0.58	-2.69 (-12.14,6.77)
Current ≥20	0.66 (0.50,0.87)	0.003	-29.32 (-49.23,-9.4)

*Adjusted for age, gender, calendar year, ethnicity (first 40 principal components), recruitment centre and smoking status. Adjusted and unadjusted incidence rate ratios are reported in table S1.
**Adjusted for pack-years, age, gender, calendar year, ethnicity (first 40 principal components), recruitment centre in overall analysis of regular smokers.

Table S6: The genetically instrumented cross-sectional relationships between bilirubin and potential mediating/intervening variables in the relationship with lung cancer.

Total	377,294	OR (95%CI) per 5 µmol/L increase*	p-value
Family history of lung cancer			
Overall	48,615 (12.9%)	0.99 (0.97 to 1.00)	0.095
Never smokers	25,338 (12.3%)	1.00 (0.98 to 1.03)	0.90
Former smokers	17,825 (13.4%)	0.97 (0.95 to 1.00)	0.079
Current smokers	5,241 (13.8%)	0.94 (0.89 to 0.99)	0.031
History of COPD/emphysema			
Overall	8,627 (2.3%)	1.04 (0.00 to 1.08)	0.03
Never smokers	2,427 (1.2%)	1.07 (0.99 to 1.15)	0.076
Former smokers	4,155 (3.1%)	1.03 (0.97 to 1.10)	0.31
Current smokers	1,992 (5.2%)	1.06 (0.97 to 1.15)	0.19

*Adjusted for age, gender, calendar year, ethnicity (first 40 principal components) recruitment centre and smoking status. Estimates derived using a one-sample Mendelian randomisation approach and the two-stage predictor substitution method.

Table S7: Associations between genetically instrumented serum total bilirubin and all-cause mortality and cancer mortality.

	Events	Person Years	Incidence rate (95%CI)	Adjusted IRR *per 5 µmol/L increase* (95%CI)*	Predicted incidence change in 10,000 PYs *per 5 µmol/L increase* (95%CI)*	p-value
All-cause mortality						
Overall	15258	328	46.5 (45.7,47.2)	0.98 (0.95,1.01)	-1.08 (-2.49,0.33)	0.13
Never smokers	5676	180	31.6 (30.8,32.4)	0.98 (0.93,1.02)	-0.86 (-2.56,0.85)	0.32
Former smokers	6471	115	56.2 (54.9,57.6)	0.98 (0.94,1.03)	-1.01 (-3.28,1.26)	0.39
Current smokers	3012	33	92.1 (88.9,95.4)	0.97 (0.91,1.04)	-2.89 (-9.95,4.16)	0.42
Prefer not to report	99	1	89.2 (73.3,109.7)			
Regular smokers (cigarettes per day)**						
Overall	7168	99	72.7 (71,74.4)	0.95 (0.91,1.00)	-3.55 (-6.84,-0.27)	0.034
Former 1-19	1496	32	47 (44.6,49.4)	0.93 (0.84,1.02)	-3.70 (-8.36,0.95)	0.12
Former *≥20*	3135	42	75.3 (72.8,78)	0.95 (0.89,1.02)	-3.23 (-7.79,1.34)	0.17
Current 1-19	1137	14	82.3 (77.6,87.2)	1.04 (0.94,1.16)	4.88 (-6.7,16.46)	0.41
Current *≥20*	1145	9	133.3 (125.8,141.3)	0.91 (0.81,1.02)	-15.96 (-35.96,4.03)	0.12
Cancer mortality						
Overall	8524	328	26.0 (25.4,26.5)	0.96 (0.92,0.99)	-1.20 (-2.26,-0.14)	0.027
Never smokers	3296	180	18.4 (17.7,19.0)	0.99 (0.93,1.06)	-0.17 (-1.43,1.09)	0.79
Former smokers	3629	115	31.5 (30.5,32.6)	0.92 (0.86,0.98)	-2.31 (-4.11,-0.51)	0.012
Current smokers	1551	33	47.4 (45.1,49.8)	0.96 (0.87,1.05)	-2.45 (-7.59,2.69)	0.35
Prefer not to report	48	1	43.3 (32.6,57.4)			
Regular smokers (cigarettes per day)**						
Overall	3949	99	40.0 (38.8,41.3)	0.92 (0.87,0.98)	-3.37 (-5.85,-0.9)	0.0075
Former 1-19	890	32	27.9 (26.2,29.8)	0.91 (0.80,1.04)	-2.53 (-6.14,1.08)	0.17
Former *≥20*	1732	42	41.6 (39.7,43.6)	0.90 (0.82,0.99)	-4.04 (-7.58,-0.49)	0.025
Current 1-19	609	14	44.1 (40.7,47.7)	1.02 (0.89,1.18)	1.15 (-7.10,9.41)	0.78
Current *≥20*	580	9	67.5 (62.2,73.2)	0.92 (0.79,1.08)	-7.08 (-20.82,6.66)	0.312

IRR=incidence rate ratio; PY=person years

*Adjusted for age, gender, calendar year, ethnicity (first 40 principal components), recruitment centre and smoking status. Participants preferring not to report smoking status were excluded due to low events.

**Adjusted for pack-years in overall analysis of regular smokers. Participants currently smoking less than 1 cigarette per day at recruitment are excluded from the smoking sub-categories but included in the overall analysis of regular smokers if they had formerly smoked one or more per day and it was possible to calculate pack-years.

Horsfall LJ, *et al. Thorax* 2020; 75:955–964. doi: 10.1136/thoraxjnl-2020-214756

References

1. Bycroft C, Freeman C, Petkova D, et al. Genome-wide genetic data on ~500,000 UK Biobank participants. *bioRxiv* 2017:166298. doi: 10.1101/166298
2. Harrell FE. Regression modeling strategies : with applications to linear models, logistic regression, and survival analysis. New York: Springer 2001.
3. Royston P. marginscontplot: Plotting the marginal effects of continuous predictors. *Stata Journal* 2013;13(3):510-27.
4. Burgess S, Thompson SG, Burgess S. Mendelian randomization : methods for using genetic variants in causal estimation. Boca Raton, FL: CRC Press, Taylor & Francis Group 2015.
5. Wain LV, Shrine N, Miller S, et al. Novel insights into the genetics of smoking behaviour, lung function, and chronic obstructive pulmonary disease (UK BiLEVE): a genetic association study in UK Biobank. *Lancet Respir Med* 2015;3(10):769-81. doi: 10.1016/S2213-2600(15)00283-0 [published Online First: 2015/10/02]
6. Weber S. bacon: An effective way to detect outliers in multivariate data using Stata (and Mata). *Stata Journal* 2010; 10(3). http://ageconsearch.umn.edu/record/159017/files/sjart_st0197.pdf (accessed 2010).
7. Brown KF, Rumgay H, Dunlop C, et al. The fraction of cancer attributable to modifiable risk factors in England, Wales, Scotland, Northern Ireland, and the United Kingdom in 2015. *Br J Cancer* 2018;118(8):1130-41. doi: 10.1038/s41416-018-0029-6 [published Online First: 2018/03/24]
